# Screening for Posttraumatic Stress Symptoms in Young Refugees: Comparison of Questionnaire Data with and without Involvement of an Interpreter

**DOI:** 10.3390/ijerph18136803

**Published:** 2021-06-24

**Authors:** Lauritz Rudolf Floribert Müller, Johanna Unterhitzenberger, Svenja Wintersohl, Rita Rosner, Julia König

**Affiliations:** Department of Psychology, Catholic University Eichstätt-Ingolstadt, Ostenstraße 25, 85072 Eichstätt, Germany; johanna.unterhitzenberger@ku.de (J.U.); svenja.wintersohl@gmx.de (S.W.); rita.rosner@ku.de (R.R.); julia.koenig@ku.de (J.K.)

**Keywords:** refugee, adolescent, assessment, screening, PTSD, interpreter

## Abstract

**Background**: The substantial number of young refugees who have arrived in Europe since 2015 requires rapid screening to identify those in need of treatment. However, translated versions of screening measures are not always available, necessitating the support of interpreters. The Child and Adolescent Trauma Screen (CATS) is a validated questionnaire for posttraumatic stress symptoms. Here, we report on the psychometric properties of the CATS in a sample of young refugees as a function of interpreter involvement. **Methods:** A total of *N* = 145 (*M*_age_ = 16.8, *SD* = 1.54; 93% male) were assessed with the CATS, with half of the screenings conducted with and half without interpreters. Post hoc analyses included calculating internal consistency using Cronbach’s *α*. We used confirmative factor analysis to investigate the factor structure. **Results:** The CATS total scale showed good reliability (α = 0.84). Differences in psychometric properties between the interpreter vs. the no interpreter group were minor and tended to be in favor of the interpreter group. Results of a confirmatory factor analysis were acceptable after the exclusion of items with low item-scale correlations. **Conclusions:** The sample and the administration of the assessment represent the situation of young refugees in Germany, where resources are low and translated versions not always available. The CATS may be a helpful screening tool for clinicians working with young refugees, even when administered with an interpreter. Limitations include the post hoc design of the analysis without randomization of participants and the lack of a third comparison group using translated questionnaire versions.

## 1. Introduction

Asylum-seeking youth, and especially unaccompanied asylum-seeking youth, have a high risk for posttraumatic stress disorder (PTSD; [1,2]). A brief and valid screening should constitute the first step of recently proposed stepped-care models to meet the psychological needs of young refugees, which are currently being tested in this group [3]. To this end, measures must be validated for culturally diverse samples. The Child and Adolescent Trauma Screen (CATS; [4]) is a short self-report questionnaire with items corresponding to PTSD criteria in the Diagnostic and Statistical Manual for Mental Disorders, 5th edition (DSM-5; [5]). It has been validated in an international (Western) sample with good reliability and validity and has since been used repeatedly with refugee youth [1,6,7].

However, self-report screening measures, including the CATS, are not available for all languages of interest in the refugee context, and, moreover, many asylum-seeking youth are illiterate and cannot use translated versions even when they exist [8]. Accordingly, in real-life care settings, screenings are often conducted with non-native language versions even when the respondents’ language proficiency is low or must be supported by interpreters. In this context, untrained lay interpreters, who might be unfamiliar with mental health services, are often employed [9]. In principle, it is conceivable that the involvement of (lay) interpreters could change both screening results and therapeutic processes for a variety of reasons, including omission of cultural taboos in the translation process, role difficulties that might lead interpreters to take a directive rather than a neutral role, and lack of professional understanding regarding mental health services [10]. In terms of therapy effectiveness, the few existing post hoc findings on interpreter involvement present a mixed picture [11,12], whereby the latest study found that the group with interpreters involved showed poorer results than the comparison group without interpreters [12]. However, there are very few quantitative findings on the effects of employing an interpreter in mental health settings in general and, to our knowledge, no studies on the effects on screening or diagnostic results in particular.

Accordingly, this post hoc study aims (1) to investigate the internal consistency of the CATS in a sample of young refugees resettled to Germany using German language versions with vs. without the involvement of an interpreter; (2) to compare symptom severity, item values and item-scale correlations between the two groups; and (3) to examine the factor structure of the CATS in the total sample.

## 2. Materials and Methods

### 2.1. Procedure and Participants

Data from two studies were used with a total of *N* = 145. Study 1 assessed the mental health of *n* = 98 minor refugees resettled in southern Germany in an interview-like setting [1]. Study 2 was a pilot study [6] examining trauma-focused cognitive behavioral therapy [13] with *n* = 47 unaccompanied refugee minors. Overall, participants were, on average, *M* = 16.73 years of age (*SD* = 1.54). Most participants were unaccompanied (*n* = 115, 78.8%) and boys (*n* = 135, 93.1%), reflecting the population structure in Germany, where most unaccompanied refugee minors are male (for further sociodemographic information, see Supplementary Material Table S1).

Both studies were approved by the IRB of the Catholic University Eichstätt-Ingolstadt (2016/23 and 2015/02/16) and participants gave their written informed consent to participate in the respective study.

### 2.2. Measure

The Child and Adolescent Trauma Screen (CATS) assesses posttraumatic stress symptoms (PTSS) according to DSM-5 in youngsters aged 7 to 17 years [4]. Participants indicate whether they have experienced 15 potentially traumatic events (yes/no) and specify the currently most distressing event. Then, they rate 20 items on PTSS on a 4-point Likert scale (0—*never* to 3—*almost always*). The sum score is between 0 and 60, with scores ≥21 indicating clinically relevant PTSS. The four subscales—intrusion, avoidance, negative alterations in cognition and mood (NACM), and hyperarousal—can be formed, which refer to the PTSD diagnostic criteria in the DSM-5. The CATS has been validated in English, German, and Norwegian language versions. We used the German version. In half of the sample, *n* = 72 (50%), interpreters supported diagnostics (for one Study 1 participant, information on interpreter involvement was missing).

## 3. Data Analysis

We used SPSS version 25 (IBM, Armonk, USA) for all analyses. We examined descriptive statistics and internal consistency (Cronbach’s *α*) for the CATS total score and symptom subscales, and for cases with and without involvement of an interpreter. We computed *χ^2^*-statistics to examine differences in internal consistency between the two subgroups using cocron [14]. A confirmative factor analysis was applied to investigate the previously confirmed DSM-5 factor structure using AMOS for SPSS version 25. For RMSEA, values ≤ 0.06 (marginal: 0.07–0.08) [15] and for CFI and TLI, values ≥ 0.95 (marginal: 0.90–0.94) indicate a good fit [16].

## 4. Results

### 4.1. Internal Consistency

The CATS showed a good internal consistency with Cronbach’s *α* = 0.84 in the total sample. Internal consistency was slightly, but not significantly, higher when assessed with an interpreter (*n* = 72; *α* = 0.85) than without one (*n* = 72; *α* = 0.82; *χ^2^*(1) = 0.684, *p* = 0.408). The four symptom clusters differed in their internal consistency and showed rather poor results (see Table 1). Internal consistencies of the four subscales were consistently, but not significantly, lower in the group without an interpreter, with avoidance even having a negative value in this group.

### 4.2. Symptom Scores and Item Analyses

Participants from Study 1 had lower average CATS scores, *M* = 22.40, *SD* = 9.35, than participants from Study 2, *M* = 26.87, SD = 10.35, *F* (143) = 2.61, *p* = 0.010. CATS scores did not vary with interpreter involvement in either subsample. In Study 1, cases without interpreters had a mean CATS score of *M* = 21.56, *SD* = 9.07, and cases with interpreters of *M* = 23.91, *SD* = 9.78, *T* (96) = -1.200, *p* = 0.233, and in Study 2, cases without interpreters had a mean CATS score of *M* = 29.33, *SD* = 7.14 and with interpreters *M* = 26.51, *SD* = 11.03, *T* (44) = 0.727, *p* = 0.471.

Means and standard deviations, as well as corrected item-scale correlations of the 20 CATS items, are given in Table 2. Five items in the sample without interpreters, and three items in the sample with interpreters had item-scale correlations ≤ 0.30, resulting in five items (8, 10, 12, 16, and 17) in the overall sample with item-scale correlations ≤ 0.30.

### 4.3. Factorial Validity

The four-factor CFA model proposed by the DSM-5 and found by Sachser et al. (2017) did not show a very good fit. While RMSEA was good, at 0.06 (90% CI 0.04–0.07), the CFI was, at 0.86, below the acceptable range. Removing five items with item-scale correlations at or below *r* = 0.30 resulted in a slightly worse, but still acceptable, RMSEA of 0.07 (90% CI 0.05–0.09), and improved CFI to 0.89 (see Table 2).

## 5. Discussion

We reported on the post hoc psychometric evaluation of the CATS as a function of interpreter involvement in a sample of severely traumatized refugee youngsters from 17 countries. The CATS previously showed good psychometric properties with translated versions and samples from Germany, Norway, and the US [4]. In our sample, the overall internal reliability was lower, but still good and did not differ between the subgroup that involved an interpreter and the one that did not. In our sample, however, avoidance, hyperarousal, and NACM showed questionable to very poor alphas, with a negative α for avoidance in the group without an interpreter. It must be kept in mind, however, that this subscale has only two items dealing with different forms of avoidance and is, therefore, not ideal to begin with from a factor analytic point of view [17]. Although the internal consistencies of the subscales tended to be consistently better in the group with interpreters, some of them were still outside the acceptable range. On the one hand, this could indicate that the young refugees were unfamiliar with psychological test formats [18], that language difficulties might have occurred, or that culturally divergent concepts were captured by the questionnaires. On the other hand, the sometimes low reliability even in the interpreter group may also stem from the difficulty of translating almost simultaneously in the screening situation and the resulting quality of translation for culturally disputable constructs. Accordingly, translated questionnaire versions, in which the translation process is more elaborate due to iterative improvements, could contribute to increasing the reliability in the recording of the subscales [19].

Five items had low corrected item-scale correlations of 0.30 or below, and this problem was more pronounced in the group not supported by an interpreter. The four-factor model showed an acceptable fit only after removing items with bad item characteristics and even then, fit indices were worse than those reported in the original study [4]. Given the heterogeneity of our sample including refugee youth from 17 countries, this deviation from the original study, which examined more homogeneous national samples, may not, however, be too surprising.

### 5.1. Limitations

A key limitation of this analysis is its retrospective nature and the fact that the variable of interest is difficult to manipulate experimentally. Study clinicians decided whether to employ an interpreter for screening according to their perception of participants’ German language skills and preferences. However, most studies on the impact of interpreters on mental health services share this limitation. While sample 1 offered voluntary participation in a screening study, sample 2 was a service use sample, which might have resulted in selection biases affecting this analysis. Furthermore, a third group using translated versions was lacking and the subsamples were too small so as to conduct CFAs for both groups separately.

### 5.2. Implications

Overall, the psychometric differences between the two groups were small and tended to favor the assistance of interpreters (internal consistency, item-scale correlations). Considering the multilingual population of young refugees in Germany, where translated versions of screening measures are often lacking, the common practice to employ lay interpreters does not seem to be disadvantageous, at least in terms of reliability.

## 6. Conclusions

The CATS is a reliable screening instrument for PTSS in culturally diverse refugee youth samples, including its use with the involvement of interpreters, making it a feasible screening tool for professionals working in this field (available at https://ulmer-onlineklinik.de/course/view.php?id=1701, accessed on 24 June 2021). Assessments should be followed by a (semi-)structured interview to ascertain clinically relevant symptoms or diagnostic status. From a scientific point of view, these findings should be verified by means of a validation of the CATS with a gold standard clinical interview. Given the paucity of quantitative research findings on the influence of interpreters on processes in mental health services, which has been emphasized by a variety of research groups [10,20], this study can be understood as a starting point for further rigorous studies, despite the limitations due to the post hoc design.

## Figures and Tables

**Table 1:** Internal consistencies (Cronbach’s α) of the four subscales in the full sample and the two subsamples.

	Full Sample	Interpreter	No Interpreter	Group Difference	
	(*n* = 145)	(*n* = 72)	(*n* = 72)	*χ^2^*(1)	*p*
**CATS total**	0.838	0.852	0.818	0.684	0.408
**Intrusion**	0.727	0.748	0.699	0.370	0.543
**Avoidance**	0.310	0.432	−0.029	1.813 ^a^	0.178
**NACM**	0.659	0.656	0.654	<0.001	0.983
**Hyperarousal**	0.585	0.606	0.580	0.052	0.821

Note. CATS = Child and Adolescent Trauma Screen. NACM = Negative alterations in cognition and mood. ^a^ As it was not possible to compute the comparison with a negative value of α, α = 0 was substituted in the group without interpreter.

**Table 2:** Item means, standard deviations and corrected item-scale correlations in the full sample and the two subsamples.

	Full Sample (*n* = 145)			Interpreter (*n* = 72)			No Interpreter (*n* = 72)		
Item	*M*	*SD*	ISC	*M*	*SD*	ISC	*M*	*SD*	ISC
1. Upsetting thoughts or pictures about what happened that pop into your head.	1.61	0.96	0.45	1.67	0.99	0.42	1.56	0.93	0.48
2. Bad dreams reminding you of what happened.	1.37	0.96	0.40	1.38	0.94	0.45	1.35	0.98	0.37
3. Feeling as if what happened is happening all over again.	0.99	1.05	0.47	1.19 ^a^	1.11	0.50	0.81 ^a^	0.96	0.41
4. Feeling very upset when you are reminded of what happened.	1.73	0.95	0.52	1.85	0.94	0.54	1.63	0.94	0.48
5. Strong feelings in your body when you are reminded of what happened (sweating, heart beating fast, upset stomach).	1.37	1.03	0.50	1.44	1.05	0.49	1.28	1.02	0.52
6. Trying not to think about what happened. Or to not have feelings about it.	2.00	0.90	0.37	2.14	0.92	0.52	1.88	0.85	**0.17**
7. Staying away from anything that reminds you of what happened (people, places, things, situations, talks).	1.05	1.11	0.38	1.32 ^b^	1.17	0.33	0.79 ^b^	0.99	0.40
*8. Not being able to remember part of what happened.*	0.70	0.89	**0.13**	0.74	0.87	**0.15**	0.67	0.92	**0.10**
9. Negative thoughts about yourself or others. Thoughts like I will not have a good life, no one can be trusted, the whole world is unsafe.	1.25	1.12	0.51	1.29	1.14	0.60	1.22	1.10	0.39
*10. Blaming yourself for what happened. Or blaming someone else when it is not their fault.*	0.67	0.87	**0.30**	0.69	0.87	0.33	0.65	0.87	**0.25**
11. Bad feelings (afraid, angry, guilty, ashamed) a lot of the time.	1.43	0.94	0.56	1.49	1.03	0.57	1.38	0.85	0.55
*12. Not wanting to do things you used to do.*	1.14	1.14	**0.30**	1.17	1.15	**0.27**	1.11	1.15	0.33
13. Not feeling close to people.	0.86	0.98	0.43	0.94	1.05	0.43	0.78	0.91	0.42
14. Not being able to have good or happy feelings.	1.15	1.00	0.57	1.24	1.05	0.61	1.07	0.95	0.52
15. Feeling mad. Having fits of anger and taking it out on others.	1.03	0.96	0.45	1.06	1.01	0.43	1.01	0.93	0.48
*16. Doing unsafe things.*	0.33	0.64	**0.29**	0.26	0.60	0.44	0.40	0.66	**0.16**
*17. Being overly careful (checking to see who is around you).*	1.23	1.12	**0.17**	1.19	1.11	**0.14**	1.26	1.14	**0.22**
18. Being jumpy.	1.11	1.05	0.43	1.03	1.01	0.39	1.19	1.10	0.52
19. Problems paying attention.	1.12	1.00	0.52	1.32 ^c^	1.06	0.59	0.93 ^c^	0.91	0.41
20. Trouble falling or staying asleep.	1.72	1.13	0.55	1.85	1.16	0.58	1.57	1.09	0.53

Note. The subscales are composed as follows: Intrusion (items 1, 2, 3, 4, 5), avoidance (items 6, 7), NACM (items 8, 9, 10, 11, 12, 13, 14), and hyperarousal (items 15, 16, 17, 18, 19, 20). *M* = mean; *SD* = standard deviation; ISC = item-scale correlations. ISCs ≤ 0.30 are in bold print. ^a^ significant difference between means, *T* (139.1) = −2.251, *p* = 0.026. ^b^ significant difference between means, *T* (138.1) = −2.915, *p* = 0.004. ^c^ significant difference between means, *T* (138.8) = −2.364, *p* = 0.019.

## Data Availability

The datasets generated and analyzed during this study are not publicly available due to sensitive and potentially identifying participant information but are available from the corresponding author on reasonable request.

## Supplementary Materials

The following are available online at https://www.mdpi.com/article/10.3390/ijerph18136803/s1, Table S1: Characteristics of study sample.

## Abbreviations

CATS	Child and Adolescent Trauma Screen
CFA	Confirmatory factor analysis
CFI	Comparative fit index
DSM-5	Diagnostic and Statistical Manual for Mental Disorders, 5th edition
NACM	Negative alterations in cognition and mood
RMSEA	Root mean square error of approximation
PTSD	Post-traumatic stress disorder
PTSS	Post-traumatic stress symptoms
SPSS	Statistical Package for the Social Sciences
TLI	Tucker–Lewis index
