# Supplementary material for: Screening for Posttraumatic Stress Symptoms in Young Refugees: Comparison of Questionnaire Data with and without Involvement of an Interpreter
Source: Int J Environ Res Public Health. 2021 Jun 24;18(13):6803. doi: 10.3390/ijerph18136803 (PMC8297356; doi:10.3390/ijerph18136803)
Supplement: Supplementary file 1 [file ijerph-18-06803-s001.zip › Supplementary Material.pdf]

International Journal of
Environmental Research and Public Health

MDPI

Supplementary Material

Table S1. Characteristics of study sample.

	**Total Sample (*N* = 145)**	**Study 1 (*n* = 98)**	**Study 2 (*n* = 47)**
	n (%) or *M* (*SD*)	*n* (%) or *M* (*SD*)	*n* (%) or *M* (*SD*)
**Age**	16.75 (1.54)	16.28 (1.69)	17.16 (0.95)
**Gender**			
female	10 (6.9%)	10 (10.2%)	0 (0%)
male	135 (93.1%)	88 (89.8%)	45 (100%)
**Country of Origin**			
Afghanistan	82 (56.6%)	54 (55.1%)	28 (62.2%)
Syria	18 (12.4%)	14 (14.3%)	3 (6.7%)
Eritrea	14 (9.7%)	11 (11.2%)	3 (6.7%)
Iraq	9 (6.2%)	8 (8.2%)	1 (2.2%)
Gambia	4 (2.8%)	3 (3.1%)	1 (2.2%)
Somalia	4 (2.8%)	2 (2.0%)	2 (4.4%)
Albania	2 (1.4%)	1 (1.0%)	1 (2.2%)
Iran	2 (1.4%)	1 (1.0%)	1 (2.2%)
Sudan	2 (1.4%)	0 (0%)	1 (2.2%)
Bangladesh	1 (0.7%)	1 (1.0%)	0 (0%)
Ethiopia	1 (0.7%)	1 (1.0%)	0 (0%)
Ivory Coast	1 (0.7%)	0 (0%)	1 (2.2%)
Libya	1 (0.7%)	0 (0%)	1 (2.2%)
Mali	1 (0.7%)	1 (1.0%)	0 (0%)
Pakistan	1 (0.7%)	1 (1.0%)	0 (0%)
Senegal	1 (0.7%)	0 (0%)	1 (2.2%)
Sierra Leone	1 (0.7%)	0 (0%)	1 (2.2%)
**Time in Germany (months)**	18.11 (8.43)	21.46 (7.73)	11.19 (4.82)
**School attendance (years)**	5.99 (3.17), range 0–12	5.90 (2.93), range 0–11	6.00 (3.67), range 0–12
**Unaccompanied**	115 (78.8%)	68 (69.4)	45 (100%)
**Number of Traumatic Event Types**	7.99 (2.56)	7.68 (2.55)	8.60 (2.51)
**CATS Symptom Score**	23.76 (9.92)	22.40 (9.35)	26.73 (10.56)

Note. *M* = mean; *SD* = standard deviation.
